# The Digital Distribution of Public Health News Surrounding the Human Papillomavirus Vaccination: A Longitudinal Infodemiology Study

**DOI:** 10.2196/publichealth.3310

**Published:** 2015-03-18

**Authors:** L Meghan Mahoney, Tang Tang, Kai Ji, Jessica Ulrich-Schad

**Affiliations:** ^1^ West Chester University West Chester, PA United States; ^2^ University of Akron Akron, OH United States; ^3^ University of New Hampshire Durham, NH United States; ^4^ Purdue University West Fayette, IN United States

**Keywords:** new media, public health dissemination, health communication, social media, HPV vaccination, infodemiology, infoveillance

## Abstract

**Background:**

New media changes the dissemination of public health information and misinformation. During a guest appearance on the *Today Show*, US Representative Michele Bachmann claimed that human papillomavirus (HPV) vaccines could cause “mental retardation”.

**Objective:**

The purpose of this study is to explore how new media influences the type of public health information users access, as well as the impact to these platforms after a major controversy. Specifically, this study aims to examine the similarities and differences in the dissemination of news articles related to the HPV vaccination between Google News and Twitter, as well as how the content of news changed after Michele Bachmann’s controversial comment.

**Methods:**

This study used a purposive sampling to draw the first 100 news articles that appeared on Google News and the first 100 articles that appeared on Twitter from August 1-October 31, 2011. Article tone, source, topics, concerns, references, publication date, and interactive features were coded. The intercoder reliability had a total agreement of .90.

**Results:**

Results indicate that 44.0% of the articles (88/200) about the HPV vaccination had a positive tone, 32.5% (65/200) maintained a neutral tone, while 23.5% (47/200) presented a negative tone. Protection against diseases 82.0% (164/200), vaccine eligibility for females 75.5% (151/200), and side effects 59.0% (118/200) were the top three topics covered by these articles. Google News and Twitter articles significantly differed in article tone, source, topics, concerns covered, types of sources referenced in the article, and uses of interactive features. Most notably, topic focus changed from public health information towards political conversation after Bachmann’s comment. Before the comment, the HPV vaccine news talked more often about vaccine dosing (*P*<.001), duration (*P*=.005), vaccine eligibility for females (*P*=.03), and protection against diseases (*P*=.04) than did the later pieces. After the controversy, the news topic shifted towards politics (*P*=.01) and talked more about HPV vaccine eligibility for males (*P*=.01).

**Conclusions:**

This longitudinal infodemiology study suggests that new media influences public health communication, knowledge transaction, and poses potential problems in the amount of misinformation disseminated during public health campaigns. In addition, the study calls for more research to adopt an infodemiology approach to explore relationships between online information supply and public health decisions.

## Introduction

The dissemination of health communication has undergone dramatic changes as digital distribution and social media transfer considerable power to users and enhance opportunities for asynchronous mass delivery. According to the Pew Internet & American Life Project [1], 72% of Internet users seek health information online. Thus, a critical public health concern is the quality of health information consumed and disseminated on the Web [2,3]. This study examines how digital distribution and social media impact the diversity of the public health information gathering and dissemination process. Through an investigation of a specific public health initiative—the human papillomavirus (HPV) vaccination—a better understanding is gained of how online health news is presented, as well as the influence that structure has on information dissemination.

HPV represents one of the most common sexually transmitted infections linked to cancer. Researchers found that an underestimated 93% of invasive cervical cancers worldwide contain HPV [2]. The Food and Drug Administration (FDA) approved vaccinations that prevent certain HPV infections and reduce the incident of cervical cancer and other anogenital cancers. Medical organizations and professionals, including the World Health Organization and Advisory Committee on Immunization Practices (ACIP), praise this HPV vaccination and encourage coverage for all females [3-5]. It is also important to note that although the HPV vaccination was originally recommended for young females, the ACIP has begun recommending it for boys as well in 2011 [5]. Nonetheless, since its introduction, dialogue surrounding the HPV vaccination has raised concerns due to its recommended administration to young girls, making it a highly controversial public health debate.

While health professionals argue for stronger public health campaigns to promote the HPV vaccination, communication efforts have been challenged on political platforms by US Representative Michele Bachmann. During a guest appearance on the *Today Show* on September 13, 2011*,* after criticizing Texas Governor Rick Perry, GOP Presidential candidate, for mandating HPV vaccines for school girls, Bachmann claimed that a crying woman had recently approached her and said that her daughter received the HPV vaccine and consequently developed “mental retardation” [6]. The American Academy of Pediatrics (AAP) responded by saying “There is absolutely no scientific validity to this statement”. Nonetheless, online news and dialogue surrounding the topic of HPV vaccination began linking to the misinformation in Bachmann’s comment.

The distribution of this type of public health misinformation is unclear and proves especially difficult to track due to unlimited diverse online news sources and the ability of social media users to participate and negotiate the information-exchange process. Public health specialists suggest that new media, such as social networking sites and online news aggregators, may have the potential to impact the public’s understandings and their adoption decisions of the HPV vaccination [3,5]. There is clearly a need to understand Internet dialogue and dissemination surrounding public health issues further, particularly ones that are surrounded by such a public health controversy.

Eysenbach [7,8] suggests that an infodemiology approach can help measure information diffusion and knowledge transaction and provide valuable insights to health professionals when misinformation happens. Infodemiology is defined as “the science of distribution and determinants of information in an electronic medium, specifically the Internet, or in a population, with the ultimate aim to inform public health and public policy [8]”. Thus, this study uses the infodemiology approach to explore the usage of online media as a new space of public health information gathering and dissemination. Specifically, it examines news diffusion of the HPV vaccination by comparing stories distributed on Google News and “retweets” distributed on Twitter, before and after Bachmann’s controversial comment.

### Challenges of the Human Papillomavirus Vaccination Initiative

The Centers for Disease Control (CDC) reports that as of June 2011, more than 35 million doses of the HPV vaccination had been distributed in the United States. Nonetheless, the introduction of HPV vaccination has come with its own unique set of challenges. One key challenge in the promotion of the HPV vaccination program is the recommended age of immunization. The recommendation is to vaccinate young girls prior to sexual debut, with the recommendation of ages starting at 11-12 years [9]. Additionally, the social stigma attached to HPV as a sexually transmitted disease may prevent the highest risk population from receiving vaccinations [10]. Parents have voiced concerns over the sexual implications of HPV vaccination leading towards earlier or higher-risk sexual activities [9-11]. Moreover, the HPV vaccination requires a round of three shots for vaccination protection. Research shows that just 38.2% of girls who received the first vaccination complete all three vaccine doses in the recommended timeframe of 365 days [12]. This suggests that even if a patient understands the benefits and opts in to the HPV vaccination, the majority will not complete the immunization process. There is a need for increasing health communication regarding the dosing and duration of the vaccination. Furthermore, the target population for the HPV vaccination program is difficult to reach through traditional public health messages [3]. For these reasons, innovative efforts are needed to educate parents and young women about the benefits of cervical cancer prevention.

New technologies allow greater opportunities for difficult-to-reach patients to receive health information and make personal health care decisions [13]. However, the frequency of misinformation online may actually negatively impact the public’s health decisions. Indeed, controversies about vaccine’s side effects have long been a challenge for public health professionals. As early as in the 1990s, incorrect dialogue linked autism disorders and the measles-mumps-rubella (MMR) vaccine, which caused significant drops in vaccination rates and increased incidence of disease [14]. It is evident that such a controversy about the side effects of a vaccine may prevent treatment. Levine [11] demonstrates how much of the public dialogue surrounding the HPV vaccination focuses on common misconceptions regarding HPV, specifically that it causes Guillain-Barré Syndrome and leads to muscle weakness and paralysis. This misnomer can then be repeated and shared across unlimited media platforms without scientific sources or credible information. Therefore, it is important to use infodemiology metrics to follow the online dialogue and news dissemination surrounding the HPV vaccination.

### News Coverage of the Human Papillomavirus Vaccination

Many scholars have examined the news coverage of the HPV vaccination when it was first released [10,11,14-17]. Johnson et al [15] examined newspaper articles on the HPV vaccination for 19 months after the FDA approved the first HPV vaccine and found the press coverage lacked detailed health information. Few mentioned the dosing, duration, effectiveness, and/or side effects of the vaccination. Wardle et al [16] examined the news coverage of the HPV vaccination in the United Kingdom and found British newspapers had a positive tone towards the vaccine in general. However, increasing risky sexual behavior has been a major topic discussed by the press. CDC researchers [10] also found that vaccine affordability was the most often-mentioned concern about the HPV vaccination among 250 search engine articles that they analyzed. Online news stories disseminated a more balanced tone regarding the vaccine. Nakada et al [17] found that the national agreement on HPV vaccination in Japan positively contributed to the advocacy of vaccine beneficiaries through media coverage online and in print.

While scholars provided important insights on how media first disseminate information related to the HPV vaccination, little scholarly attention has been given to social media dissemination reports, which could be crucial to the vaccination promotion today.

This type of follow-up research becomes even more necessary when additional news surrounding the vaccination has little to do with the public health initiative itself. For example, in September 2011, US Representative Michele Bachmann brought the side effects of the HPV vaccines into the political discussion. Her controversial comment that HPV vaccination could cause mental retardation soon fueled online debate between scientific, political, and family communities [6,14,18]. Even though the AAP issued an official response, reiterating that Bachmann’s assertion was false, media coverage of this response was not as widely disseminated as Bachmann’s controversial comment itself [14].

While it is challenging to examine how much influence this type of public health misinformation has on personal health care decisions, it is valuable to investigate information shared through online public forums. “Although few would argue that spreading blatant misinformation should be a punishable offense, false claims about vaccine risk can have deadly consequences when they discourage vaccination” [14]. This calls for a more thorough longitudinal infodemiology study of how controversy impacts online information dissemination surrounding the HPV vaccination.

**Figure 1:**
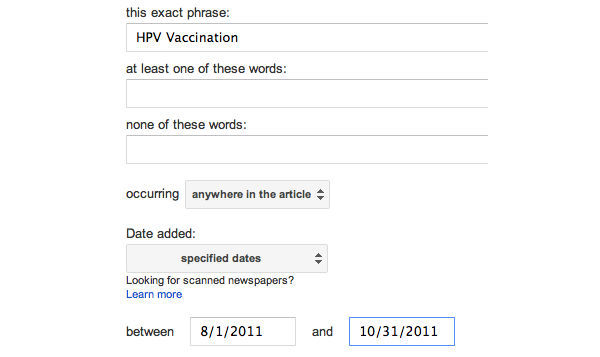
Google News criteria.

**Figure 2:**
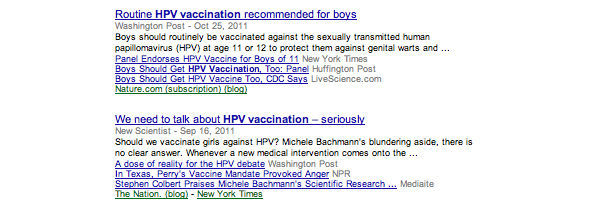
Google News sample articles.

**Figure 3:**
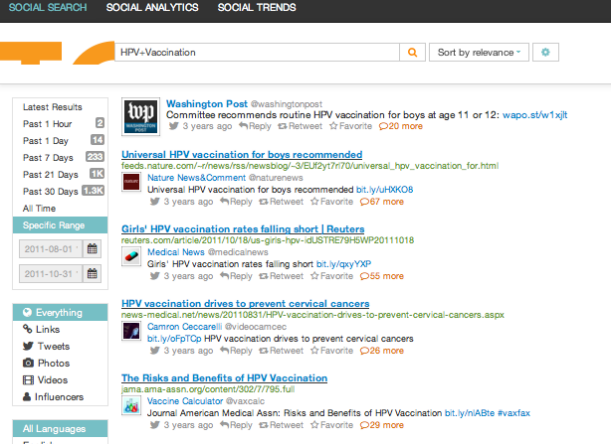
Topsy search criteria and sample articles.

The purpose of this study is to compare aggregated news stories shared on Google News and retweets shared on Twitter both before and after Bachmann’s comment to help understand the public health information dissemination through both social media and news aggregates. A snapshot of the Google News search criteria is illustrated in Figure 1; a sample of this search is found in Figure 2. A snapshot of the Twitter search criteria and results is found in Figure 3. Specifically, this research aims to answer the following research questions:

RQ1: What are the similarities and differences in the HPV vaccination coverage between Google News and Twitter?

RQ2: How did the content of news articles centered on the HPV vaccination change after Bachmann’s 2011 interview on the *Today Show*?

## Methods

### Sample and Unit Analysis

Google News and Twitter are among the primary sources that researchers use to study news media and information dissemination [19-21]. Google News is a computer-generated news aggregator that provides readers news articles from over 4500 sources, making it one of the most popular sources for online news [22,23]. Google News offers links to the news articles based on the criteria provided by the users (eg, subject, timeline), as well as the characteristics of news content such as relevance and “how often and on what sites a story appears online” [22]. Through Google News Archive Advanced Search, users are able to find news articles on any subject during any time period.

Twitter, on the other hand, is a social media site that provides information, news, and communication about what users are interested in [24,25]. On Twitter, individuals disseminate information or news via “tweets”, which are short messages each within 140-character limit. Twitter users are able to tweet about any topic and to provide links to full stories via abbreviated dialogue. One of the most popular features of Twitter is the ability for users to “retweet” stories they find in other users’ feeds. Research points to this retweeting option as a primary tool for disseminating information about important news topics [21].

While it may be argued that the structure and purpose of news dissemination on Twitter differs from that on Google News, it is appropriate to analyze and compare articles disseminated through both platforms. Social media research demonstrates how Twitter is being used primarily as a resource for individuals to access and communicate on recent news issues [19,20]. Furthermore, public health literature examines Twitter as a platform to investigate important health care news dissemination [21- 23]. Thus, this research used news articles disseminated by Google News and news articles linked to “retweets” on Twitter as materials to study information dissemination of HPV vaccination.

Google News Archive database was used to identify Google News articles on HPV vaccination from August 1-October 31, 2011. The 3-month time period was chosen because it includes 1.5 months before and 1.5 months after Bachmann’s comment about the HPV vaccines on September 13, 2011. In addition, a 3-month period is a common timeframe used by content analysis studies on information dissemination [26,27]. Topsy database was used to identify news articles linked to retweets on Twitter regarding HPV vaccination. Topsy is a partner of Twitter that analyzes billions of tweets on a daily basis and provides one of the largest public indexes of social media posts [28]. Similar to Google News search, Topsy users can find news articles retweeted on Twitter on any subject during any time period. The same time period (ie, August 1-October 31, 2011) was used in the Topsy search. The search term “HPV vaccination” was entered into both Google News search and Topsy search.

A purposive sampling was used to draw the first 100 news articles that appeared on the Google News search results pages and the first 100 news articles that appeared on the Topsy search results pages. This represents a sample that audiences are likely to access and read, as previous research suggests that users typically start from the top of search results/tweets and read only the first few pages of search results/tweets [8,29]. Duplication was not used in this research. Duplicated articles were recorded only once to increase variety and the degree of representativeness within the sample.

### Coding

Each news article was coded for seven key variables: (1) date (ie, before or after Bachmann’s comment), (2) tone of the article (ie, positive, neutral, or negative), (3) article source (ie, government website, health organization website, newspaper/magazine, portal site, radio program transcript, scientific journal, TV program transcript, user-generated content, or other), (4) topics covered in the article (ie, vaccine efficacy, dosing, duration, protection against diseases, side effects, cost, politics, vaccine eligibility for females, and vaccine eligibility for males), (5) concerns raised in the article (ie, increasing sexual risk behavior, mandatory school vaccination, importance of continued Pap smears, age concern, safety, accessibility, and affordability), (6) types of sources referenced in the article (ie, medical doctors, political and government officials/organizations, CDC, vaccine manufacturer representatives, cancer organizations, research community, personal accounts, and other), and (7) interactivity (ie, hyperlink, search function, comment, and share). All the coding categories are based on previous literature [30,31].

Two researchers carried out the coding independently. Coders were trained using a preliminary subset of news stories. The training process continued until the coders were comfortable with the various coding categories [32]. Detailed definitions of categories were provided for coding. All of the selected news articles were downloaded to a computer hard drive for the purpose of coding and conducting an intercoder reliability test, as Google News and Twitter frequently update their pages.

Approximately 20% of the total sample was randomly selected to assess intercoder reliability, including news articles disseminated by both Google News and Twitter. A Cohen’s kappa test was run on all seven variables that required a judgment call from the coders. The measure of agreement was as follows: date=1.0, tone of the article=.85, article source=.98, topics covered in the article=.90, concerns raised in the article=.80, types of sources referenced in the article=.78, and interactivity=1.0. The total agreement was .90, which indicates a high level of reliability on the coding instrument and across coders.

## Results

A total of 100 Google News articles and 100 Twitter articles were coded to examine the similarities and differences in information gathering and diffusion of HPV vaccination. Overall, 37.5% articles (75/200) were published before Bachmann’s comment, and 62.5% (125/200) appeared after her interview. Specifically, among the 100 articles from Google News, 45.0% (45/100) were published before Bachmann’s comment, and 55.0% (55/100) were after. Among the Twitter articles coded in this study, 30.0% (30/100) were published before Bachmann’s interview, while 70.0% (70/100) were after.

Among the news articles analyzed in this study, 44.0% (88/200) had a positive tone towards HPV vaccination, 32.5% (65/200) a neutral tone, and 23.5% (47/200) presented a negative tone. The top three topics covered by these articles were protection against diseases (82.0%, 164/200), vaccine eligibility for females (75.5%, 151/200), and side effects (59.0%, 118/200). Safety (68.5%, 137/200), mandatory school vaccination (44.5%, 89/200), and age concern (30.0%, 60/200) were the most frequently mentioned concerns related to the vaccine. In addition, CDC (46.5%, 93/200) was the most-cited reference in these articles, followed closely by the research community (45.0%, 90/200), and medical doctors (40.5%, 81/200). Most of the news articles disseminated by Google News and Twitter were newspaper/magazine articles (44.0%, 88/200), articles appearing on health organizations’ websites (17.0%, 34/200), and user-generated content (14.5%, 29/200). Furthermore, this analysis found that increased interactivity became a trend during this public health dialogue. The majority of the news articles coded in this study included more than one type of interactive feature, such as hyperlinks and search functions.

To answer RQ1, this study found that Google News and Twitter articles significantly differed in tone, source, topics, concerns raised, types of sources referenced, and uses of interactive features. First, results indicate that news articles presented by Google News and linked to Twitter presented different tones when diffusing information about the HPV vaccination. Among the Twitter articles coded in this study, 54.0% (54/100) had a positive tone, 29.0% (29/100) a neutral tone, while only 17.0% (17/100) had a negative tone. On the other hand, Google News had an equal distribution in terms of the article tone towards HPV vaccination, that is, positive (34.0%, 34/100), neutral (36.0%, 36/100), negative (30.0%, 30/100). The chi-square test result was significant (χ^2^_2_=8.895, *P*=.01; see Table 1). Findings suggest that Twitter disseminated more “positive” articles related to the HPV vaccines compared to its Google counterparts, while Google News presented more “neutral” and “negative” pieces than did Twitter.

**Table 1:** Article tones used by Twitter and Google News HPV vaccination articles.

Tones	Twitter (n=100)		Google News (n=100)	
	n	%	n	%
Positive	54	54.0	34	34.0
Neutral	29	29.0	36	36.0
Negative	17	17.0	30	30.0

In addition, this study found that Google News and Twitter were significantly different in their coverage about vaccine eligibility for females (χ^2^_1_=9.758, *P*=.002; see Table 2), politics (χ^2^_1_=7.788, *P*=.005), protection against diseases (χ^2^_1_=6.640, *P*=.01), side effects (χ^2^_1_=4.051, *P*=.04), and vaccine efficacy (χ^2^_1_=3.945, *P*=.047). Specifically, Google News articles covered topics of vaccine eligibility for females, protection against diseases, and vaccine efficacy significantly more frequently than its Twitter counterparts. On the contrary, news articles linked to Twitter-reported topics around politics and side effects significantly more often than did Google News. No statistically significant differences were found in coverage about vaccine cost, duration, dosing, and eligibility for males between news articles disseminated by Google News and Twitter. Protection against diseases (89.0%, 89/100) was the most frequently covered topic by Google News, followed by vaccine eligibility for females (85.0%, 85/100) and vaccine efficacy (53.0%, 53/100). The top three topics for Twitter articles were protection against diseases (75.0%, 75/100), side effects (66.0%, 66/100), and vaccine eligibility for females (66.0%, 66/100).

Vaccine accessibility was the only concern expressed significantly differently between Google News and Twitter articles. Articles linked to Twitter addressed the accessibility of the HPV vaccination more frequently than did Google News (χ^2^_1_=14.624, *P*<.001). No statistically significant differences were found regarding any other concerns coded in this study. Safety (63.0%, 63/100), mandatory school vaccination (38.0%, 38/100), and age concerns (32.0%, 32/100) were the most frequently mentioned concerns regarding the HPV vaccination in Google News articles. Similarly, safety (74.0%, 74/100), mandatory school vaccination (51.0%, 51/100), and affordability (30.0%, 30/100) were the top three concerns raised by articles linked to Twitter.

**Table 2:** Topics covered by Twitter and Google News HPV vaccination articles.

Topics	Twitter (n=100)		Google News (n=100)		χ^2^	df	*P* value
	Freq.	%	Freq.	%			
Protection against diseases	75	75.0	89	89.0	6.640^b^	1	.01
Side effects	66	66.0	52	52.0	4.051^a^	1	.04
Vaccine eligibility for females	66	66.0	85	85.0	9.758^b^	1	.002
Duration	48	48.0	41	41.0	0.992	1	.32
Politics	46	46.0	27	27.0	7.788^a^	1	.005
Dosing	39	39.0	33	33.0	0.781	1	.38
Vaccine efficacy	39	39.0	53	53.0	3.945^b^	1	.047
Vaccine cost	33	33.0	29	29.0	0.374	1	.54
Vaccine eligibility for males	31	31.0	27	27.0	0.389	1	.53

^a^Covered more frequently by the Twitter articles (*P*<.05).

^b^Covered more frequently by the Google News articles (*P*<.05).

As shown in Table 3, this study found that Google News and Twitter significantly differed in the references they used when disseminating news regarding HPV vaccination. Twitter articles used personal accounts (χ^2^_1_=16.860, *P*<.001), medical doctors (χ^2^_1_=5.996, *P*=.01), and political and government officials/organizations (χ^2^_1_=4.119, *P*=.04) as reference sources significantly more often than its Google counterparts; while Google News cited the research community more frequently than did Twitter (χ^2^_1_=5.172, *P*=.02). No statistically significant differences were found in the quotations from CDC, vaccine manufacturers, and cancer organizations. CDC (52.0%, 52/100), medical doctors (49.0%, 49/100), and political officials (46.0%, 46/100) were the most frequently used references in Twitter articles, while the research community (53.0%, 53/100) was the major reference for Google News articles, followed by CDC (41.0%, 41/100). It is also important to note that while 31.0% (31/100) of the Twitter articles used personal accounts as a reference source, only 8.0% (8/100) of the articles from Google News did the same.

**Table 3:** Types of sources referenced by Twitter and Google News HPV vaccination articles.

Reference sources	Twitter (n=100)		Google News (n=100)		χ^2^	df	*P* value
	Freq.	%	Freq.	%			
CDC	52	52.0	41	41.0	2.432	1	.12
Medical doctors	49	49.0	32	32.0	5.996^a^	1	.01
Political/Government officials	46	46.0	32	32.0	4.119^a^	1	.04
Research community	37	37.0	53	53.0	5.172^b^	1	.02
Personal accounts	31	31.0	8	8.0	16.860^a^	1	<.001
Cancer organizations	28	28.0	27	27.0	0.025	1	.87
Vaccine manufacturer	8	8.0	10	10.0	0.244	1	.62
Other	4	4.0	5	5.0	0.116	1	.73

^a^Used more frequently by the Twitter articles (*P*<.05).

^b^Used more frequently by the Google News articles (*P*<.05).

Moreover, newspapers/magazines (33.0%, 33/100), user-generated content (28.0%, 28/100), and health organizations’ websites (19.0%, 19/100) were the top three sources for articles linked to Twitter, while newspapers/magazines (55.0%, 55/100), health organizations’ websites (15.0%, 15/100), and scientific journals (11.0%, 11/100) were the major sources for Google News articles. Interestingly, while 4.0% (4/100) of the Twitter articles used government websites as the source to disseminate information about the HPV vaccines, zero Google News articles came from government websites. While 28.0% (28/100) of the Twitter articles about the vaccination came from user-generated content, only one Google News article (1.0%, 1/100) used this source. The chi-square test suggests a significant difference in article source used by Google News and Twitter (χ^2^_8_=39.997, *P*<.001; see Table 4).

**Table 4:** Article sources used by the Twitter and Google News HPV vaccination articles.

Information sources	Twitter (n=100)		Google News (n=100)	
	Freq.	%	Freq.	%
Newspapers/Magazines	33	33.0	55	55.0
User-generated content	28	28.0	1	1.0
Health organization websites	19	19.0	15	15.0
Scientific journals	7	7.0	11	11.0
TV	6	6.0	10	10.0
Government websites	4	4.0	0	0.0
Other sources	2	2.0	4	4.0
Portal sites	1	1.0	2	2.0
Radio	0	0.0	2	2.0

While this study found that both articles from Google News and those linked to Twitter encouraged the interactivity between content providers and consumers during this public dialogue about HPV vaccination, significantly more Twitter articles allowed users to leave comments, compared to its Google News counterparts (χ^2^_1_=16.262, *P*<.001; see Table 5). On the other hand, articles from Google News included search functions and hyperlinks significantly more often than did the Twitter ones (χ^2^_1_=19.175, *P*<.001; and χ^2^_1_=6.133, *P*=.01 respectively).

**Table 5:** Interactive features used by Twitter and Google News HPV vaccination articles.

Interactivity	Twitter (n=100)		Google News (n=100)		χ^2^	df	*P* value
	Freq.	%	Freq.	%			
Comments	89	89.0	65	65.0	16.262^a^	1	<.001
Share	88	88.0	92	92.0	0.889	1	.35
Hyperlinks	77	77.0	90	90.0	6.133^b^	1	.01
Search functions	67	67.0	92	92.0	19.175^b^	1	<.001

^a^Used more frequently by the Twitter articles (*P*<.05).

^b^Used more frequently by the Google News articles (*P*<.05).

To answer RQ2, this study tests the similarities and differences in news articles regarding HPV vaccination before and after Michele Bachmann’s *Today Show* interview. Topic shift was the major change that happened. As shown in Table 6, the news articles covered six out of the nine topics coded in this study significantly differently before and after Bachmann’s remark. Before Bachmann’s comment, the news surrounding HPV vaccination focused on vaccine dosing (χ^2^_1_=13.333, *P*<.001), duration (χ^2^_1_=8.002, *P*=.005), vaccine eligibility for females (χ^2^_1_=4.687, *P*=.03), and protection against diseases (χ^2^_1_=4.372, *P*=.04) significantly more often than did the later pieces. After Bachmann’s *Today Show* appearance, not surprisingly, the news topics shifted towards politics (χ^2^_1_=6.456, *P*=.01), and interestingly, news articles talked about HPV vaccine eligibility for males more frequently (χ^2^_1_=6.223, *P*=.01).

Specifically, Twitter articles covered information regarding the HPV vaccine dosing (before: 67%, 20/30; after: 27%, 19/70; χ^2^_1_=13.789, *P*<.001), duration (before: 73%, 22/30; after: 37%, 26/70; χ^2^_1_=11.020, *P*=.001), eligibility for females (before: 83%, 25/30; after: 59%, 41/70; χ^2^_1_=5.738, *P*=.02), and protection against diseases (before: 90%, 27/30; after: 69%, 48/70; χ^2^_1_=5.143, *P*=.02) significantly more often before Bachmann’s interview than did the later pieces. Nonetheless, after Bachmann’s *Today Show* appearance, Twitter linked to more HPV news articles focused on politics than before (before: 23%, 7/30; after: 56%, 39/70; χ^2^_1_=8.864, *P*=.003). It is important to note that this study found no significant topic changes in Google News articles before and after Bachmann’s comment.

**Table 6:** Topics covered by the HPV vaccination articles before and after Bachmann’s comment.

Topics	Before (n=75)		After (n=125)		χ^2^	df	*P*
	Freq.	%	Freq.	%			
Protection against diseases	67	89.3	97	77.6	4.372^a^	1	.04
Vaccine eligibility for females	63	84.0	88	70.4	4.687^a^	1	.03
Side effects	44	58.7	74	59.2	0.006	1	.94
Duration	43	57.3	46	36.8	8.002^a^	1	.005
Dosing	39	52.0	33	26.4	13.333^a^	1	<.001
Vaccine efficacy	37	49.3	55	44.0	0.537	1	.46
Vaccine cost	21	28.0	41	32.8	0.505	1	.48
Politics	19	25.3	54	43.2	6.456^b^	1	.01
Vaccine eligibility for males	14	18.7	44	35.2	6.223^b^	1	.01

^a^Covered more often by the articles published before the Bachmann’s comment (*P*<.05).

^b^Covered more often by the articles published after Bachmann’s comment (*P*<.05).

In addition, this study found that the articles focused on HPV vaccination used cancer organizations as a reference source significantly more often after Bachmann’s comment than before (χ^2^_1_=7.960, *P*=.005). However, no statistically significant differences were found in uses of other types of reference sources in these articles before and after the controversy. The top three reference sources used before Bachmann’s comment were CDC (49%, 37/75), the research community (47%, 35/75), and medical doctors (45%, 34/75); while afterwards, the top three were CDC (45%, 56/125), the research community (44%, 55/125), and political and government officials/organizations (43%, 54/125).

Specifically, Google News articles cited cancer organizations significantly more often after Bachmann’s comment than before (before: 9%, 4/45; after: 42%, 23/55; χ^2^_1_=13.616, *P*<.001). Nonetheless, Twitter articles used government and political officials/organizations as reference sources significantly more frequently after Bachmann’s comment than before (before: 23%, 7/30; after: 56%, 39/70; χ^2^_1_=8.864, *P*=.003). In terms of concerns raised in these HPV vaccine news, Twitter articles expressed concerns related to accessibility of the vaccine (before: 50%, 15/30; after: 20%, 14/70; χ^2^_1_=9.179, *P*=.002) and the importance of continued Pap smears (before: 13%, 4/30; after: 3%, 2/70; χ^2^_1_=4.086, *P*=.04) significantly more often before the comment than the later pieces. Articles from Google News mentioned concerns related to mandatory school vaccination significantly more before Bachmann’s *Today Show* appearance than its later pieces (before: 49%, 22/45; after: 29%, 16/55; χ^2^_1_=4.118, *P*=.04). This study did not find significant differences in terms of article tone, interactivity, and article source between news disseminated before and after Bachmann’s comment.

## Discussion

### Principal Findings

This longitudinal infodemiology study examined the similarities and differences in the dissemination of news articles related to the HPV vaccination between Google News and Twitter, as well as how the content of news changed after Bachmann’s controversial 2011 appearance on the *Today Show.* Overall, this study found that protection against diseases, vaccine eligibility for females, and side effects were the topics most often covered by the coded HPV vaccination articles, while safety, mandatory school vaccination, and age concern were the most widely expressed concerns. This is an alarming trend, as health communication research calls for a more focused diffusion of detailed treatment-related information about the HPV vaccines rather than concerns that may drive the public dialogue in a non-health related direction [15]. While participation in health-related websites advances personal empowerment and user satisfactions [3,30], more needs to be done to promote public health facts, not just public dialogue.

Additionally, this study found that increased interactivity became a trend during this public dialogue. Both Google News and Twitter allowed users to share the articles, leave comments, and included links and search functions, though Twitter encouraged more comments, while Google News provided more links and search functions. These interactive functions serve as tools for interpersonal communication and recommendation, which should continue to be encouraged, since personal networks are one of the most influential factors for behavior change [31].

Through a comparison of the information disseminated between Google News and Twitter, this research found that Google News had a more balanced tone towards the HPV vaccination, while Twitter had a positive tone towards the vaccine in general. Given the function that each medium serves (Google News as a search engine and Twitter as a popular social media), this result is consistent with expectations. Nonetheless, findings suggest that Twitter took on the role of a “soap box” for users to voice outrage against Bachmann’s misinformed comment after her appearance on the *Today Show* and provided an opportunity that individuals may not have received if not for social media, especially since the articles disseminated on Google News continued taking a neutral stance regarding the vaccination. This neutral tone models a more traditional role of news in the United States, as it presented two sides of an issue, American Pediatric Society versus Michele Bachmann, even if there was no scientific evidence to support one side’s claims.

This research also compared how Bachmann’s controversial comment impacted the online public dialogue about the HPV vaccination. Interestingly, the study found no differences in overall article tones, concerns raised by the news and reference sources used before and after her appearance. According to our research, topic change was the only major shift happened after Bachmann’s comment. Results indicate a turn towards a political discussion after Bachmann’s comment. Fewer news articles talked about the important treatment information about the HPV vaccines, such as vaccine dosing, duration, and protection against diseases after the controversy. This finding demonstrates that misinformation not only pushed forth an increase in articles that contained false public health information, but transformed dialogue from a public health initiative to a political debate.

It is important to note that the content of news articles disseminated through Twitter were highly impacted by Bachmann’s misinformed comment while Google News articles remained more consistent. After the controversy, Twitter had many more articles related to politics, while Google News was able to maintain the same or even increased the amount of articles centered on the science and the vaccine itself. This finding is interesting, as it points to a key difference between a strictly news aggregate (Google News) search and an aggregator within a social media platform (Twitter). While Twitter does allow for users to “retweet” news stories that they find in other users’ feeds, they are also given the option to add on to these conversations with their own viewpoints or experiences. This suggests that Twitter provides a structure that allows for users to lead public dialogue in any direction that they choose, or in this case, politics. Google News, however, served as more of a top-down dissemination of “expert” information. While there are certainly opportunities and challenges of each of these structures, in regards to misinformation, Twitter allows for users to fight back against, or increase the spread of, misinformation. This proves a much more egalitarian medium, as the everyday user is able to trend the same manner as an expert.

If more physicians utilized Twitter and/or other social media (eg, Facebook, social support groups) as a platform to both disseminate and gather news with their patients, more could be done to minimize the misinformation shared, without diluting the participatory process for individual users. For example, if an individual heard Bachmann’s comment on the *Today Show* and immediately became concerned about HPV vaccines causing mental retardation, they would likely share that concern with their social network so that they could become informed and engage in dialogue. If their physician was a part of their online social network, he or she could also add to the conversation and help facilitate the dialogue by filtering out misinformation.

### Limitations

Although this research provides valuable insights into the dissemination of public health information via new media, it is important to note the limitations of a content analysis study. This research cannot provide accurate insights into the influence or effect that this dissemination has on users. In addition, this research examined only the top news that appeared on Twitter and Google News. Future studies may consider examining all articles within the timeframe. Nonetheless, such a purposive sample (instead of studying the entire population or drawing a probability sample) represents a practical frame of the articles audiences read, which can be more valuable when seeking an understanding of how public opinion is formed. Moreover, this study focused on the news articles 1.5 months before and 1.5 months after Bachmann’s comment, which captures the short-term differences between Twitter and Google News coverage. Future research may consider gathering data within a longer timeframe to better understand how online sources impact public opinion about important health issues.

### Conclusions

Overall, this infodemiology study suggests that new media is influencing public health communication and the patient’s role in today’s dynamic communication environment [30,33]. While this shift from physician-centered treatment towards patient-centered treatment does lead to an increase in personal empowerment and user satisfaction, it also poses potential problems in the amount of misinformation disseminated during public health campaigns. By understanding how users negotiate the structures of gathering information, medical professionals, researchers, and communication specialists will be better able to understand how mainstream media and social media reproduce a consensual public information dialogue, potentially optimizing the personal health care management process. This study also calls for more research to adopt an infodemiology approach to further explore relationships between online information supply and public health decisions.

## Abbreviations

AAD: American Academy of Pediatrics
CDC: Centers for Disease Control
FDA: Food and Drug Administration
HPV: human papillomavirus
